# Neurobiology of Cognitive Remediation Therapy for Schizophrenia: A Systematic Review

**DOI:** 10.3389/fpsyt.2014.00103

**Published:** 2014-08-15

**Authors:** Anders Lillevik Thorsen, Kyrre Johansson, Else-Marie Løberg

**Affiliations:** ^1^Department of Psychosocial Science, University of Bergen, Bergen, Norway; ^2^Division of Psychiatry, Haukeland University Hospital, Bergen, Norway; ^3^Department of Biological and Medical Psychology, University of Bergen, Bergen, Norway

**Keywords:** schizophrenia, cognitive remediation therapy, cognitive training, neuroimaging, neurocognition

## Abstract

Cognitive impairment is an important aspect of schizophrenia, where cognitive remediation therapy (CRT) is a promising treatment for improving cognitive functioning. While neurobiological dysfunction in schizophrenia has been the target of much research, the neural substrate of cognitive remediation and recovery has not been thoroughly examined. The aim of the present article is to systematically review the evidence for neural changes after CRT for schizophrenia. The reviewed studies indicate that CRT affects several brain regions and circuits, including prefrontal, parietal, and limbic areas, both in terms of activity and structure. Changes in prefrontal areas are the most reported finding, fitting to previous evidence of dysfunction in this region. Two limitations of the current research are the few studies and the lack of knowledge on the mechanisms underlying neural and cognitive changes after treatment. Despite these limitations, the current evidence suggests that CRT is associated with both neurobiological and cognitive improvement. The evidence from these findings may shed light on both the neural substrate of cognitive impairment in schizophrenia, and how better treatment can be developed and applied.

## Introduction

Neurocognitive impairment is considered a core feature of schizophrenia (1, 2) and is an important predictor of functional outcomes, including social problem solving, continued daily activities after disease onset (3), life satisfaction (4), and the ability to return to work or school (5). Though neurocognitive impairment is observed in a majority of patients with schizophrenia, it should be noted that neurocognitive functioning is heterogeneous in this patient group (6, 7). Some of the early questions in CRT research were how important cognitive deficits are to functioning, what the neural and cognitive deficits of cognitive dysfunction in schizophrenia are, and if effective treatment could be developed and delivered (8). Today, some of these questions have been answered, while others currently have promising suggestions. As noted above, cognitive deficits are certainly important in many areas of life (3–5); there is now also considerable evidence for the neural substrates of these deficits [e.g., Ref. (9)], and promising treatments exist (10). This progress has been supported by the development of guidelines and standardized batteries of cognitive tests, such as the NIMH-MATRICS Consensus Cognitive Battery (MCCB) (11, 12) and other comparable batteries [e.g., Ref. (13)]. However, the issue of neurocognitive measurement is not settled, as recent evidence indicates that neuropsychological deficits may bet better conceptualized as a single construct rather than discrete domains (14, 15).

The neurobiological dimension of cognitive dysfunction was previously understood in terms of hypoactivity, primarily in frontal areas (16, 17). However, several meta-analyses have since described cognitive dysfunction as a complex interaction of selective hypo- and hyperactivity in both cortical and subcortical areas, including frontal, parietal, and limbic structures. This has been associated with dysfunctions of distributed networks related to attention, cognitive control, and working memory among others (9, 18–21). Findings of increased activity, specifically in the prefrontal cortex (PFC), cortical midline regions, parietal and temporal cortex, insula, and amygdala have also been reported (9). The increased activity may represent compensatory cognitive responses or activity associated with characteristics other than cognitive deficits, such as differing emotional experiences (9). Increased activation in schizophrenia has also been linked to inefficient processing (22–24). Progressive structural abnormalities in schizophrenia have also been reported, both in adults (25) and adolescents (26, 27), which may be an early predictor of later transition to psychosis (28). Electrophysiological abnormalities in schizophrenia include reduced amplitude for the N1 (29), P1 (30), and P300 components, as well as increased P50 gating (31, 32). Furthermore, while reduced P300 amplitude may be common to both schizophrenia and schizophrenia-like symptoms, reduced N1 amplitude may be specific to schizophrenia (33). This shows that schizophrenia is associated with deficits in both early and later processing.

Based on the evidence that cognitive impairment and dysfunctional neural activation are prevalent and clinically important aspects of schizophrenia, many researchers have addressed the need for more attention to the treatment of these aspects of schizophrenia (2, 34, 35). However, the typical pharmacotherapy in schizophrenia is not as effective as one would hope in treating cognitive impairments (36). Two large trials, the Clinical Antipsychotic Trials of Intervention Effectiveness (CATIE) (37) and the European First-Episode Schizophrenia Trial (EUFEST) (38) showed mild to moderate improvement after atypical antipsychotic treatment in long-term or first-episode schizophrenia and schizophreniform disorder, respectively. Later studies report that most antipsychotics, especially atypical antipsychotics, may at best produce mild remediation of cognitive deficits (Cohen’s *d* between 0.20 and 0.40), while domain-specific cognitive treatment effects have not been revealed (39–41). Also, in addition to the mixed evidence for generalization to increased daily functioning, the pro-cognitive effects of atypical antipsychotics may be even less when accounting for practice effects (42).

In lieu of effective pharmacological interventions for cognitive impairment, there are several promising interventions that target cognitive functioning, such as cognitive remediation therapy (CRT) (43), cognitive enhancement therapy (44), and neuroplasticity-based auditory training (45). A distinction can be made between these approaches based on their focus on either higher-level cognitive domains, such as attention, memory, and executive function or on increasing and utilizing neuroplasticity through more basic tasks, such as early perceptual processing and working memory (46). However, though such a dichotomy is useful in highlighting the differences between the approaches, it may also underestimate the similarities between them, such as the use of “errorless learning,” self-monitoring, scaffolding, and the role of motivation in mediating treatment outcome (47). These forms of therapy all provide some form of cognitive training, either using “pen-and-paper” or through computerized exercises. The role of the therapist is often to provide feedback, adjust task difficulty, and provide strategies for learning and problem solving. Current approaches vary in terms of using broad or narrow targets of cognition, where some use very specific exercises, while others use a wide variety of interventions. The approaches also differ on whether they use extensive repetitions or share focus on repetitions and daily implementation of strategies. The different forms of cognitive interventions will be referred to as CRT from here on, as the treatments share many attributes, and have similar cognitive effects (10). CRT has been reported to have a small to moderate influence on measures of global cognition (*d*s of 0.4), verbal working memory (*d*s between 0.35 and 0.5), social cognition (*d*s between 0.5 and 0.6), and daily functioning (*d* of 0.37) (10, 48, 49). Wykes et al. (10) found that the cognitive effects were durable at follow-up for cognition and also indicated that increases in functioning are better reached by adding CRT to other rehabilitation programs, and applying wider, strategic approaches rather than a focus on drill and practice. This is further supported in a recent study, which provided combined treatment of CRT and sociocognitive training (50).

Since CRT may affect behavioral measures of cognitive processing, it is reasonable to expect underlying neurobiological changes as well. The relation between neurocognitive performance and its neural substrates may also be influenced by other variables such as clinical symptoms, motivation, and pharmacological treatment. The use of neuroimaging in CRT research may indicate whether neurocognitive changes are reflected in neural changes and vice versa. Alternatively, they may indicate that though behavioral changes are present, they do not reflect measurable changes in the brain. Since the first exploratory studies on the neurobiology of CRT (51–53), more recent and larger studies have emerged, highlighting the need for a systematic review to map the current findings and guide future research.

The aim of the present review was to systematically examine the existing research on the neurobiological effects of cognitive remediation in schizophrenia, by focusing on studies using functional and structural neuroimaging measures before and after CRT. A positive effect of CRT on the activity and integrity of brain networks previously indicated in schizophrenia was expected.

## Method

Relevant studies were identified through a systematic search of the Embase, PsycInfo, and Medline databases. Manual reference searches of relevant articles and studies were also used. The initial systematic search aimed to encompass many forms of neuroimaging, and therefore, included the terms “functional magnetic resonance imaging (fMRI),” “magnetic resonance imaging,” “positron emission tomography,” “magnetic resonance spectroscopy,” “electroencephalography (EEG),” “diffusion tensor imaging (DTI),” “near-infrared spectroscopy (NIRS),” and “magnetoencephalography (MEG).” Inclusion criteria were that studies had to use a form of cognitive remediation or training for cognitive impairment, a measurement of neural activity to investigate potential neural changes, and that all included patients were adults.

## Results

We identified 13 studies published from 2002 to 2014. Seven studies used fMRI, one used structural MRI, three used MEG, one used EEG, and one used NIRS. No studies using PET or MRS were identified. Table 1 describes the characteristics of each study, listed by date of publication.

**Table 1:** **Summary of the reviewed studies**.

Authors	Participants	Experimental task	Treatment(s)	Study design	Imaging method	Neural treatment effects	Direction of change
Wykes et al. (54)	12 SCZ 6 HC	N-back	CRT	Treatment, placebo, HC	fMRI	R inferior frontal gyrus and bilateral occipital activity	↑
Adcock et al. (55)	55 SCZ	Syllable discrimination	AT	Treatment, placebo	MEG	M1 attenuation	↑
Eack et al. (56)	53 SCZ	N/A	CET, EST	2 Treatments	MRI	Loss of GM in temporal cortex, among them the L parahippocampal gyrus, L amygdala, bilateral anterior cingulate, and L hippocampus	↓
						GM in L amygdala	↑
Haut et al. (57)	21 SCZ 9 HC	N-back, lexical task	CRT, CBSST	2 Treatments, HC	fMRI	L prefrontal activity	↑
Bor et al. (58)	20 SCZ 15 HC	N-back	CRT	Treatment, wait-list, HC	fMRI	L inferior/middle frontal gyrus, cingulate gyrus and inferior parietal lobule activity	↑
Popov et al. (59)	39 SCZ	Passive listening	AT, Cogpack	2 Treatments	MEG	M50 gating ratio	↓
Popov et al. (60)	36 SCZ	Passive listening	AT, Cogpack	2 Treatments	MEG	Gamma-band activity and alpha-band desynchronization	↑
Rass et al. (61)	44 SCZ	Passive listening	CRT	Treatment, placebo, TAU	EEG	None	
Subramaniam et al. (62)	31 SCZ 16 HC	Word generation and recognition	AT	Treatment, placebo, HC	fMRI	Medial PFC activity	↑
Penadés et al. (63)	30 SCZ 15 HC	N-back	CRT, social training	Treatment, placebo, HC	fMRI, DTI	L superior parietal lobule and bilateral middle frontal gyri activity	↑
						DMN activity in L precuneus and middle frontal gyrus	↓
						FA in CC and R posterior thalamic radiations	↑
Vianin et al. (64)	16 SCZ	Verbal fluency	CRT	Treatment, TAU	fMRI	Inferior parietal lobule, precentral gyrus, Broca’s area, middle occipital cortex, middle cingulate cortex, and superior parietal lobule activity	↑
Pu et al. (65)	31 SCZ	N-back	CRT	Treatment, TAU	NIRS	Bilateral dorsolateral PFC, left ventrolateral PFC, and right frontopolar PFC activity	↑
Subramaniam et al. (66)	30 SCZ 15 HC	N-back	AT	Treatment, placebo, HC	fMRI	Middle frontal and inferior frontal gyri activity	↑

*SCZ = schizophrenia patients; HC = healthy controls; CRT = cognitive remediation therapy; fMRI = functional magnetic resonance imaging; R = right; AT = auditory based training; EEG = electroencephalography; N/A = not applicable; CET = cognitive enhancement therapy; EST = enriched supportive therapy; MRI = magnetic resonance imaging; GM = gray matter; L = left; CBSST = cognitive behavioral social skills training; MEG = magnetoencephalography; DTI = diffusion tensor imaging; TAU = treatment as usual; NIRS = near-infrared spectroscopy*.

### fMRI and NIRS

Four of the fMRI studies primarily used *n*-back tasks as the experimental tasks as such tasks during 2-back load typically engage cognitive processes relevant to working memory. Wykes et al. (54) provided the participants in the CRT group with 40 sessions of one on one “pen-and-paper” exercises related to memory, planning, and cognitive flexibility, while another group was provided control therapy focused on relaxation and role-play. During a visual *n*-back task, the authors reported increased activation in right inferior frontal gyrus and bilateral occipital cortex for the CRT patients compared to controls. Both treatment groups also significantly differed from controls in left frontal, orbitofrontal, and right insula activity. All CRT patients improved more than 1 SD on one measure of cognitive flexibility and one dual memory span task. All nine patients used typical antipsychotics during the study, while three used atypical.

Haut et al. (57) provided 25 h of group-based computerized CRT. Using visual *n*-back tasks containing words or pictures, as well as a lexical decision task, the authors demonstrated overlapping increases in activity in the left dorsolateral PFC, anterior cingulate, left dorsal PFC, right frontopolar PFC, and left frontopolar cortex, with CRT patients displaying greater increases for all foci. CRT patients improved on word and picture 2-back *(d*s of 0.89 and 1.4, respectively), and CRT patients improved more than the control treatment group. All patients were on antipsychotics, which remained unchanged throughout treatment.

Bor et al. (58) reported that CRT patients exhibited higher levels of activation for left inferior/middle frontal gyrus, cingulate gyrus, and inferior parietal lobule/precuneus during a visual 2-back test after 28 h of computerized CRT, compared to baseline measures. CRT patients improved on measures of strategic efficiency and sustained attention *(d*s of 0.88), which was superior to wait-list controls. Patients were on stable doses of atypical antipsychotics throughout the study.

Subramaniam et al. (62) investigated how computerized cognitive training influences neural activity in regions associated with reality monitoring, using tasks of word generation and recognition of words produced by oneself or others during fMRI. Treatment consisted of either computerized cognitive training, which also included emotional identification tasks or placebo video games. Cognitive training was associated with improved source memory *(d* of 0.86 compared to placebo video games), while the placebo and healthy controls groups did not improve. The authors reported that patients exhibited less activation in the medial PFC during word-recognition for words produced by the participant at baseline, which somewhat normalized after CRT, though patients still had less activity than healthy controls. Because of the region of interest approach used by the authors, the study did not describe other possibly relevant changes in activity. Nearly all patients used atypical antipsychotics during the study.

Penadés et al. (63) investigated the effect of CRT using fMRI and DTI, providing the patients with 40 h of “pen-and-paper” CRT exercises focused on planning and working memory aided by a therapist. After treatment, decreases in activation were greater for patient who received CRT compared to social skills training. Decreased activation during a visual *n*-back task was found in the left superior parietal lobule and bilateral middle frontal gyri. Decreased activity in the default-mode network was found in the left precuneus and middle frontal gyrus, among others. Cognitive improvements on measures of verbal and non-verbal memory were small *(d*s of 0.23 and 0.38, respectively). All patients used stable doses of atypical antipsychotics during the study.

Vianin et al. (64) provided patients with 14 weeks of CRT training or treatment as usual and measured neural changes using a verbal fluency task during fMRI. For the CRT patients, the authors reported increased activation in the inferior parietal lobule, precentral gyrus, inferior frontal gyrus (Broca’s area), middle occipital cortex, middle cingulate cortex, and superior parietal lobule, compared to the control group after treatment. Neurocognitive performance also increased during Stroop *(d* of 0.45), matrix reasoning *(d* of 0.84), and Tower of Hanoi tasks *(d* of 0.34), and the CRT group increased more than the control group for all measures except for Tower of Hanoi. Nearly all patients received stable doses of atypical antipsychotics during the study.

Pu et al. (65) measured changes in prefrontal activity during *n*-back tasks using NIRS, after patients had received 60 h of computerized CRT sessions. The authors reported increased activity in bilateral dorsolateral PFC, left ventrolateral PFC, and right frontopolar PFC for the CRT group. Neurocognitive performance increased on some measures (*d* of 0.9 for verbal memory, 0.59 for executive function), but not for measures of working memory or verbal fluency. All patients used antipsychotics and seven patients in the CRT group changed daily dosage levels during the study.

Subramaniam et al. (66) presents the most recent fMRI study of CRT, using *n*-back tasks, providing 80 h of computerized auditory and sociocognitive training, compared to active videogame placebo and healthy controls. They reported baseline hypoactivation in the middle frontal gyrus. After treatment activity increased more in the middle frontal and inferior frontal gyri for the group who received auditory training, which also demonstrated superior gains in task performance. All patients remained on stable doses of antipsychotics during the study.

Functional magnetic resonance imaging is the most commonly used method of studying changes in the brain following CRT, indicating that improved cognitive performance is associated with distributed neural changes in many areas of the brain, converging in prefrontal regions.

### EEG and MEG

Adcock et al. (55) performed the very first study of MEG and CRT, which compared participants receiving auditory training for 50 h with healthy controls. They investigated the M1 response to two presented syllables, which relates to signal attenuation between the first and second presented syllables (Dale, 2010). After treatment, they found a decreased lateralization of the M1 response in the patient group, as well as changes toward normalization of the M1 (i.e., increased response in the left hemisphere, and decreased in the right). The patients also improved on measures of global cognitive functioning, verbal working memory, and learning and memory (*d*s of 0.86, 0.58, and 0.86, respectively), but not on measures of non-verbal and visual memory. Furthermore, changes in left hemisphere attenuation correlated with increases in verbal learning. The patients all remained on stable antipsychotic doses during the study.

Popov et al. (59) applied MEG to investigate the neural effects of auditory-focused cognitive exercises and computerized cognitive training (Cogpack) in relation to the M50 component, with a treatment period of 4 weeks. The M50 and P50 components underlie sensory gating, arising from the superior temporal gyrus (67), and are characterized by increased P50/M50 amplitude in schizophrenia (31). Increased M50 amplitude was also found in this study at baseline, compared to controls. However, after treatment, M50 amplitude in the auditory-focused group did not differ from controls, while P50 in the Cogpack group did not significantly change. The auditory-focused group improved more on measures of working memory *(d* of 0.80), and California Verbal Learning Test (CVLT) immediate recall (0.78), and as much as the Cogpack group on CVLT delayed recall (1.28). The patients were largely on atypical antipsychotics during the study. In another report, Popov et al. (60) found an increase in time-locked gamma-band activity (60–80 Hz) as well as increased non-time-locked alpha-band (8–12 Hz) desynchronization. Only after auditory training was alpha desynchronization associated with M50 and improved verbal memory.

Rass et al. (61) investigated cognitive and neurophysiological changes following 40 h of computerized auditory and visual training, compared to a video game placebo condition and TAU. Their results revealed no treatment effects for the P300 nor Auditory Steady State Response components, in addition to no treatment-related cognitive gains on cognitive measures. The non-significant treatment effects could be affected by the higher cognitive performance of the active placebo and TAU groups at baseline, few participants, or too low intensity in the remediation exercises.

In summary, electrophysiological studies mostly indicate that both early perceptual processes and cognitive functioning change toward normalization after CRT, though null findings have also been reported. These findings complement the functional findings using fMRI by providing evidence for early time-locked activity, compared to the slower, but more spatially accurate, BOLD response.

### MRI and DTI

The only study using structural MRI reported neuroprotective effects and potential increases in gray matter as an effect of cognitive remediation and enriched supportive therapy (EST) in a longitudinal study lasting 2 years (56). A main effect of time indicated loss of gray matter in areas such as the bilateral cerebellum, left medial, and posterior cingulate. Notably, an interaction effect of time and treatment indicated that CRT was more effective than EST for reducing gray matter loss in medial temporal areas, among them the left parahippocampal gyrus, left amygdala, bilateral anterior cingulate, and left hippocampus. The authors also reported increases in left amygdala gray matter volume for the CRT group. Neurocognitive performance increased after CRT for the for CVLT short free recall (*d* of 0.5), Trails B (*d* of 0.83), for the ratio between initiation to execution time during a Tower of London task (*d* of 1.0), where the CRT group improved more than the EST group. All patients were on stable doses of antipsychotics for the duration of the study.

Penadés et al. (63) reported increased fractional anisotropy after treatment with CRT in the body and genu of the corpus callosum and right posterior thalamic radiations using DTI. Social skills training group, however, exhibited decreased fractional anisotropy in the bilateral superior longitudinal fasciculus, and left inferior longitudinal fasciculus, further indicating how CRT may have neuroprotective effects compared to other treatments.

Though reports of structural changes are the least numerous, they provide preliminary evidence for neuroprotection, regrowth of gray matter, and increased fractional anisotropy, providing optimistic results on how progressive degradation damage may be preventable in schizophrenia.

## Discussion

The current findings of neural changes associated with CRT converge in frontal areas, including prefrontal and middle frontal areas. The studies also report changes in parietal, temporal, parahippocampal, and limbic regions. Also, neural changes were accompanied by varying improvement in neurocognitive performance, which strengthens the relation between the neurobiological and behavioral changes.

The findings suggest that CRT can positively affect processing in distributed areas, fitting to both Wykes et al. (10) findings of the somewhat wide cognitive effects of CRT, as well as Nuechterlein et al. (5) reports of dysfunction in distributed cortical and subcortical areas. The reported changes have mostly been of increased activation, while the Penadés et al. (63) finding of decreased frontal activity after treatment may be related to improvement of inefficient processing (24). The studies applying MEG and structural MRI indicate that cognitive remediation can affect temporal regions as well (56, 60). Findings of increased gating activity in parietal and temporal areas after training using MEG, and reduced gray matter loss in temporal areas using MRI also fit the proposed distributed processing network of Minzenberg et al. (9). The reduced loss, and even increases, in medial temporal gray matter reported by Eack et al. (56) indicate that CRT may prevent degradation of functions related to memory and affect.

Cingulate activation, especially of the anterior regions, is often abnormal in schizophrenia, where one meta-analysis concluded that patients had lower activity in the right anterior cingulate, and higher activity in the left anterior cingulate (9). This was partly replicated in the reviewed studies, where Haut et al. (57) reported increased right activity and Bor et al. (58) reported an increase in left cingulate activity. The anterior cingulate cortex is associated with performance monitoring and prefrontal task engagement (9).

Insula activation was found in several studies in the review, where activation was generally found to be more stable for patients than controls, and even increasing in one study (57). The insula in schizophrenia has been linked to sensory-affect processing, recognition of self- and externally generated words (68), and possibly being part of a compensatory response to reduced prefrontal processing (9). The small effect of CRT on insular activation may indicate that affective aspects are not affected to the same extent as cognitive aspects. It could be that cognitive behavior therapy (CBT) might reduce insula activation to a greater extent than CRT (69) because of CBT’s greater focus on emotional processing, although this has not been explored in current studies.

The differences between higher-level and neuroplasticity-based approaches to CRT are evident in their methodology as well as focus in treatment. Examples of this is the greater use of auditory-focused training [e.g., Ref. (45)] in the reviewed studies using EEG and MEG, which provide evidence for the effects of such treatment on early processing. Wykes et al. (10) report of less generalization of learning after neuroplasticity-based remediation receives mixed support in some of the reviewed studies, which report increased cognitive function 6 months after treatment, as well as improved occupational functioning (66), but not social functioning (62). However, though an improvement in social function was not achieved, a differential association between bilateral middle frontal gyri and occupational functioning, and medial PFC and social functioning may indicate specific neural substrates for functioning in different domains of life.

The importance of stimulating early sensory processing has received some support in current neuroimaging studies, and this is also supported by some of the reviewed studies. Some have reported increased occipital activation during task-related and resting state activity (54, 63), increased activity in Broca’s area (65), and normalized sensory gating in the superior temporal gyrus (59, 60), though changes in sensory regions are not reported in all studies. This may also be influenced by the differing usage of data-driven or *a priori* placement of regions of interest, or the use of whole-brain analysis, which may have led to the sporadic reports of neural changes in sensory regions. Notably, both studies reporting occipital changes utilized a higher-level CRT approach, which indicates that a change in early sensory processing is not exclusive to neuroplasticity-based treatment (54, 66).

The neurobiology of cognitive dysfunction in schizophrenia is complex, and has been associated with aberrant diffusion and connectivity in the corpus callosum (19–21), dysfunctional oscillations (70, 71) possibly linked to GABA (γ-aminobutyric acid), parvalbumin interneurons, NMDA (N-methyl-d-aspartate) receptors, and cortico-cortical connections (72, 73). These abnormal oscillations are found in many parts of the brain, including temporal and frontal areas. We propose that CRT may contribute to normalization of these mechanisms. CRT is associated with normalization of alpha and gamma oscillations, as reported by Popov et al. (60), as well as increased connectivity through the corpus callosum (63), which may be associated with the cortical activation found in the studies using fMRI.

The neural mechanisms that CRT acts through is not currently known, but one possibility is that the practice, guidance, and use of cognitive strategies protects remaining neurobiological and cognitive resources by strengthening the compensatory structures and activity reported by Minzenberg et al. (9), while promoting the growth and strength of new connections through neuroplasticity. This is supported by a finding of increased brain-derived neurotropic factor (BDNF) after efficacious CRT (74). As neural and cognitive abnormalities are often present before psychosis, remediation could perhaps be more effective if used at an early or prodromal stage, perhaps with at-risk children or adolescents. One study on poor readers aged 8–10 years reported promising increases in fractional anisotropy (75) after treatment, indicating the possibility of efficacious CRT even at an early age in healthy participants.

Similar effects of pro-cognitive medication and CRT may indicate common mechanisms of change. Unfortunately, few imaging studies of pharmacologically induced cognitive changes exist. Importantly, the existing studies share important limitations in the wide effects of antipsychotics on the brain, which may confound neural changes relevant to cognition with changing symptoms or side-effects (76). Relevant findings include reduced default-mode negative modulation and increased connectivity in the ventral medial PFC during *n*-back tasks (77), as well as increased activity in the sensory and ventrolateral PFC, and decreased activation in the DLPFC, striatum, and thalamus during visually guided eye saccades (78). These studies report both neural normalization and denormalization, which may be influenced by cognitive or motor side-effects of the treatments, as well as a general effect of antipsychotics on the BOLD response (76). These confounders should be considered when comparing pharmacological treatment and CRT for cognitive deficits, though they may both provide evidence for the role of prefrontal changes in cognitive improvement.

### Limitations of the current studies

Limitations of the present review include the differences in treatment strategy, outcome measures, and experimental tasks, which complicate the comparison of treatment effects. Overall, this lack of convention limits the scope of the present article. A major limitation is the number of studies included in the review, reflecting the early stages of this area of research. There are also several limitations in the reviewed articles. First, few participants in both treatment and control groups contribute to an increased risk of type II errors due to low statistical power. This can explain why the number of corresponding brain areas is not higher. Second, the long-term effects of CRT have not been explored in depth. For example, do the patients need booster sessions in order to maintain positive changes in behavior or neural activity? The findings of Eack et al. (56) showed that results remain even after 2 years after beginning treatment, though it must be taken into consideration that treatment lasted for as many as 60 sessions, while there is evidence that as few as 25 sessions (57) can mediate changes in activity. This supports the fact that CRT may have a sustained effect on neural functioning but does not serve as conclusive evidence. The reviewed studies, and CRT in general, varies in terms of treatment length and intensity. Wykes et al. (10) did report treatment length and intensity as significant predictors of outcome, and differences in these aspects may influence the neurobiology of CRT and cognitive recovery. As such, the current studies are not sufficient to answer if booster sessions or longer treatments are needed for continued neurobiological change. Another difference between the reviewed studies is the role of the therapist, which ranges from extended involvement over 40 h (54), to being purposefully minimalized (66). Though such differences may be useful in establishing specific or common factors in cognitive remediation, guidance and motivation from an experienced therapist may be useful in clinical contexts, especially for the many patients with comorbid disorders and motivational or social difficulties. Apart from this, several of the studies have weaknesses that need to be addressed. For example, several studies do not describe the randomization or blinding procedure, and some did only use single-blinding or quasi random group placement. Differences in group sizes for active treatment and healthy controls were also pervasive, which may influence the comparisons between them. The concurrent use of typical and atypical antipsychotics may confound the relation between CRT and neural changes, especially considering the mild positive cognitive effects of atypical antipsychotics. However, the similar use of these drugs in all treatment groups and studies partly controls this confounder.

## Conclusion

The beneficial effects of CRT for neurocognitive performance are established, and are often reported as being low or moderate in size. However, questions of the generalizability and cost–benefit ratio, to name a few, are still under consideration. The present article presents evidence of the beneficial neural effects of CRT, which indicates neuroplasticity through cognitive training. The use of different neuroimaging methods and treatments, though a concern with regard to direct comparison and replication, indicates that neural changes are measurable in several neuroimaging methods, and also provide preliminary evidence for normalization of early perceptual and executive processing, as well as long-lasting structural changes. There are several limitations in the current studies, the most important being that there are very few studies. Future research should seek to further investigate how and why CRT works. Potential approaches include using both pro-cognitive drugs and CRT (36, 79), or investigate the potentially common neural mechanisms of CRT, rTMS, transcranial direct current stimulation, and various pharmaceutical agents (80–83). If future studies continue to support the evidence toward cognitive and neurobiological normalization after CRT, such evidence support the extended use of CRT, as cognitive impairment is one of the greatest burdens associated with schizophrenia.

## Conflict of Interest Statement

The authors declare that the research was conducted in the absence of any commercial or financial relationships that could be construed as a potential conflict of interest.
